# Supplementary material for: Costs of Newly Funded Proton Therapy Using Time-Driven Activity-Based Costing in The Netherlands
Source: Cancers (Basel). 2023 Jan 14;15(2):516. doi: 10.3390/cancers15020516 (PMC9856812; doi:10.3390/cancers15020516)
Supplement: Supplementary file 1 [file cancers-15-00516-s001.zip › cancers-2113322-supplementary.pdf]

Supplementary Materials S1: Process map

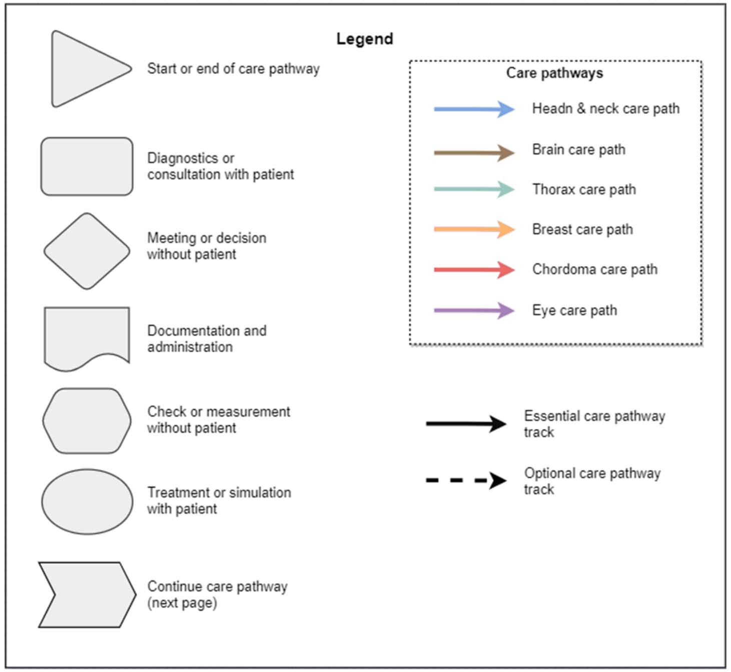

Plan comparison

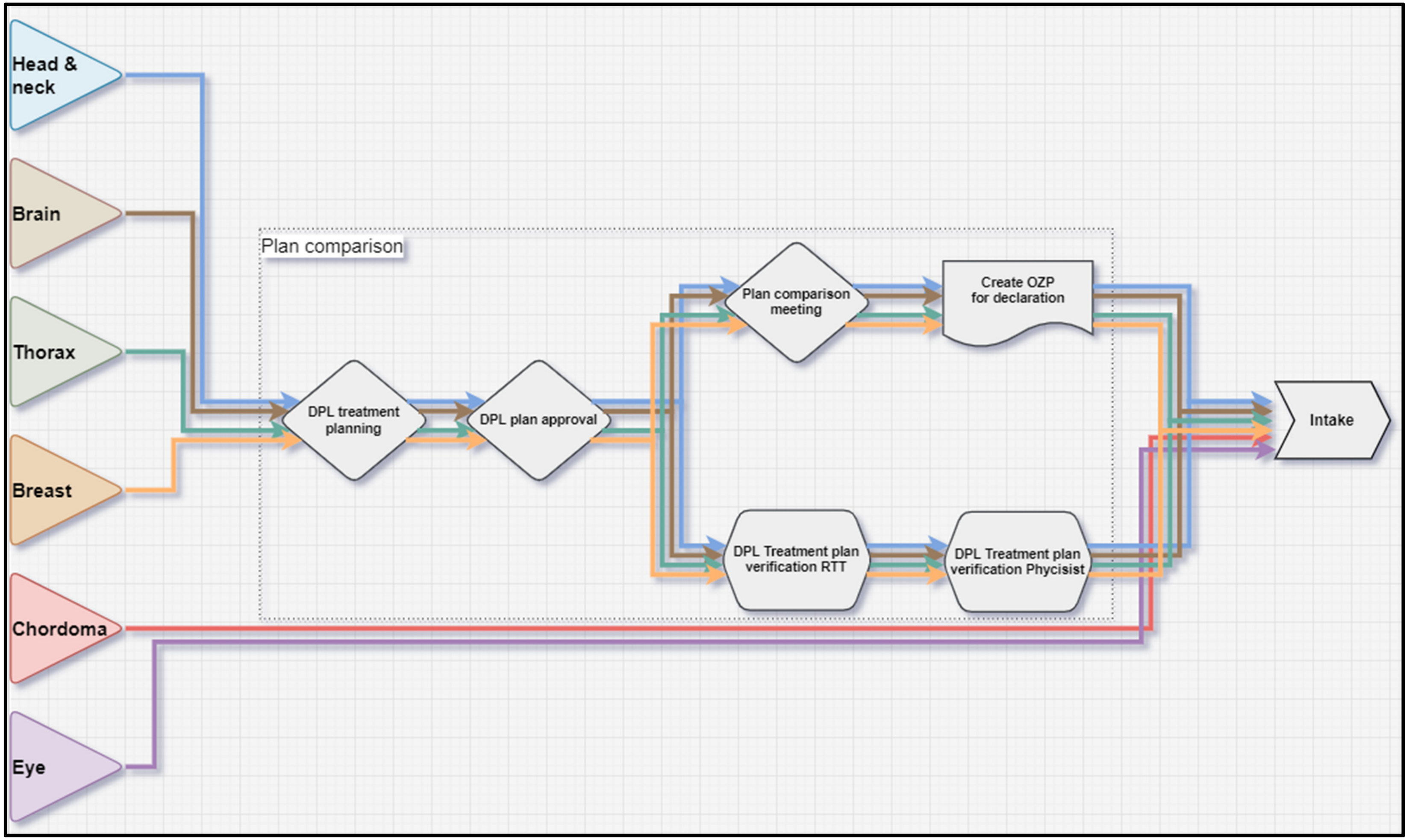

Patient intake

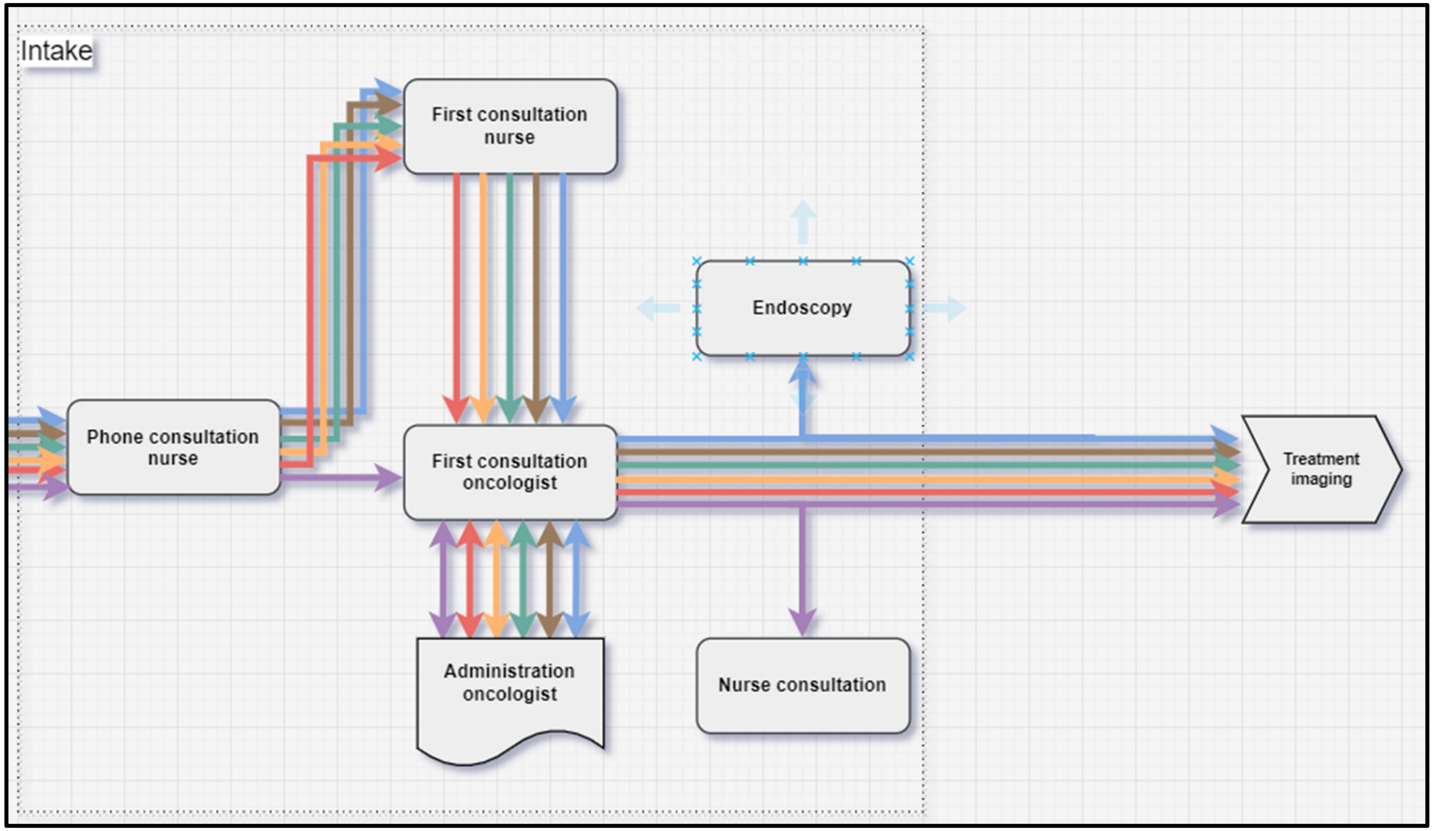

Treatment imaging

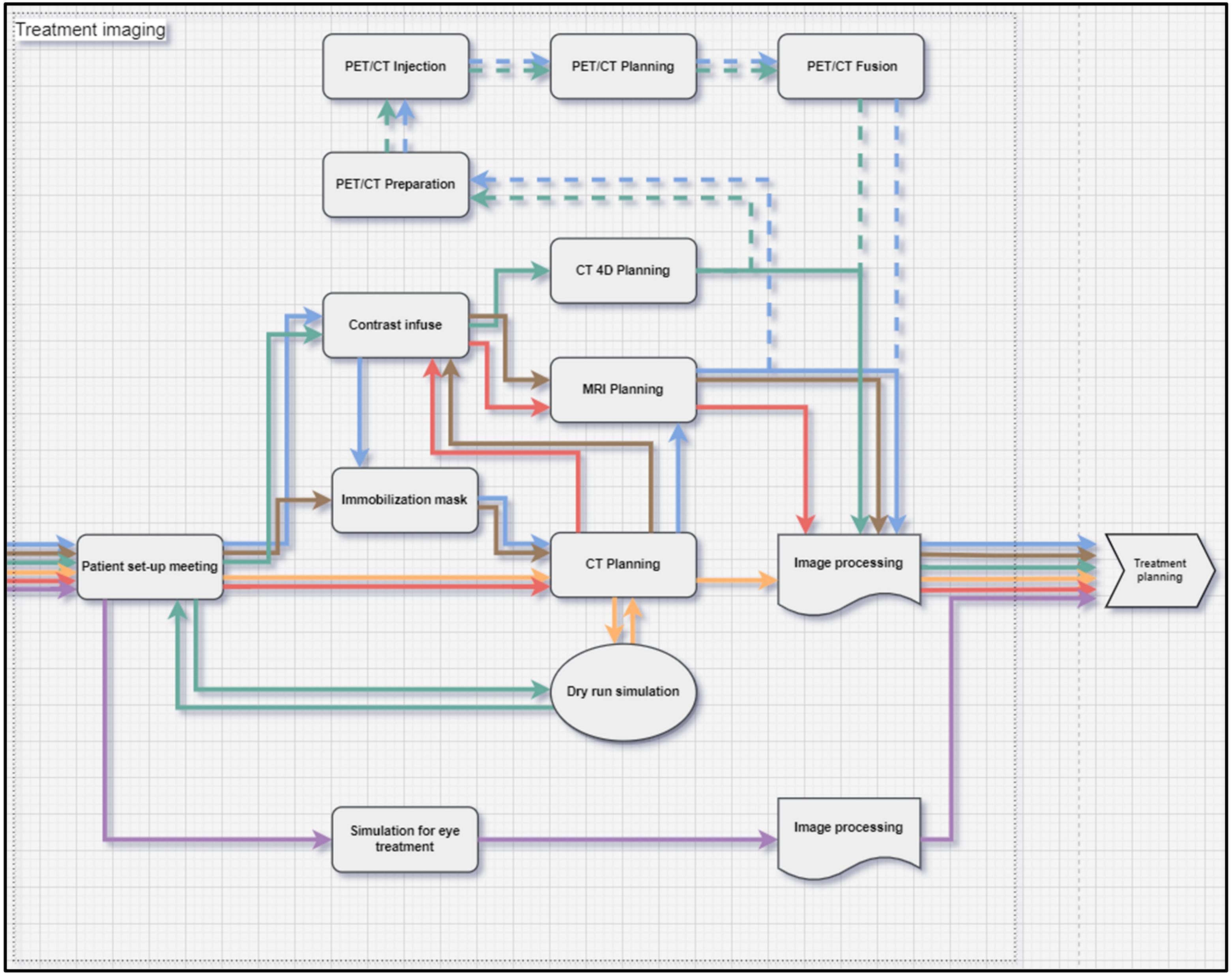

Treatment planning

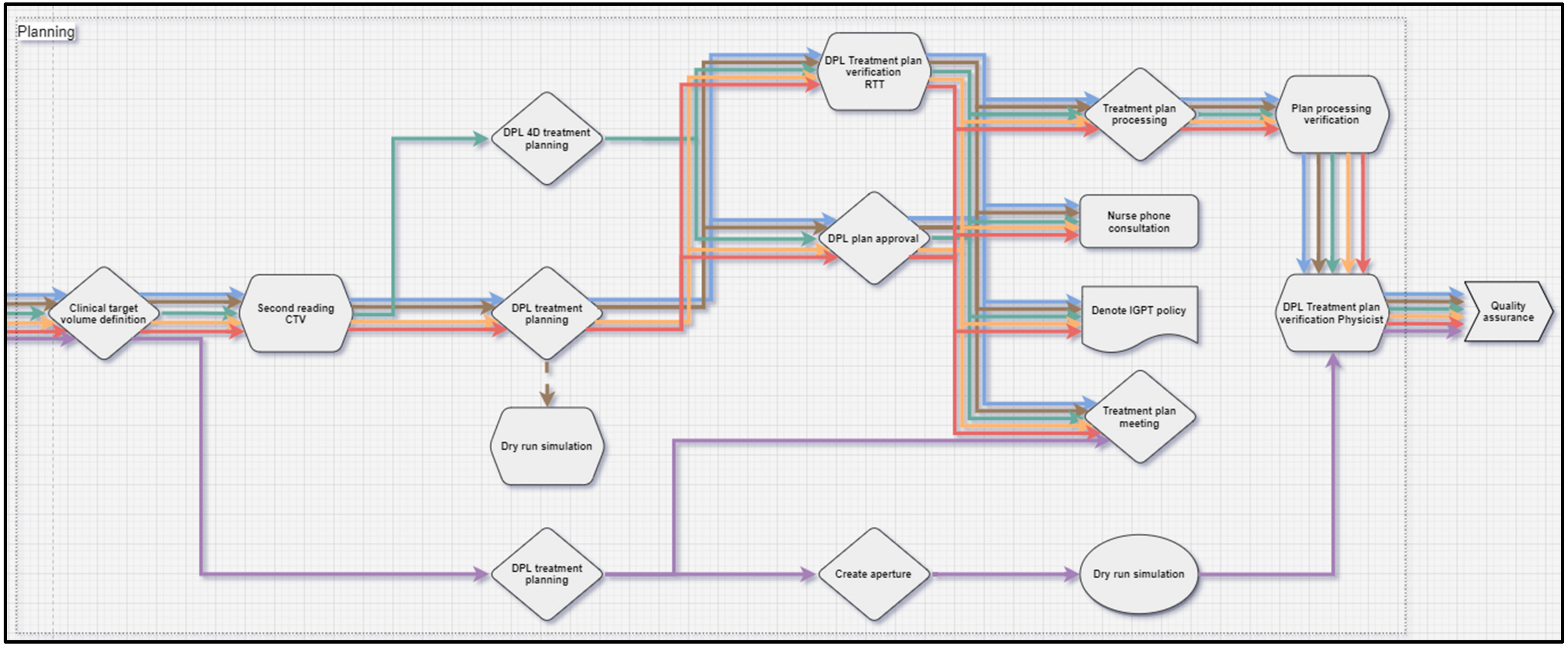

Quality assurance

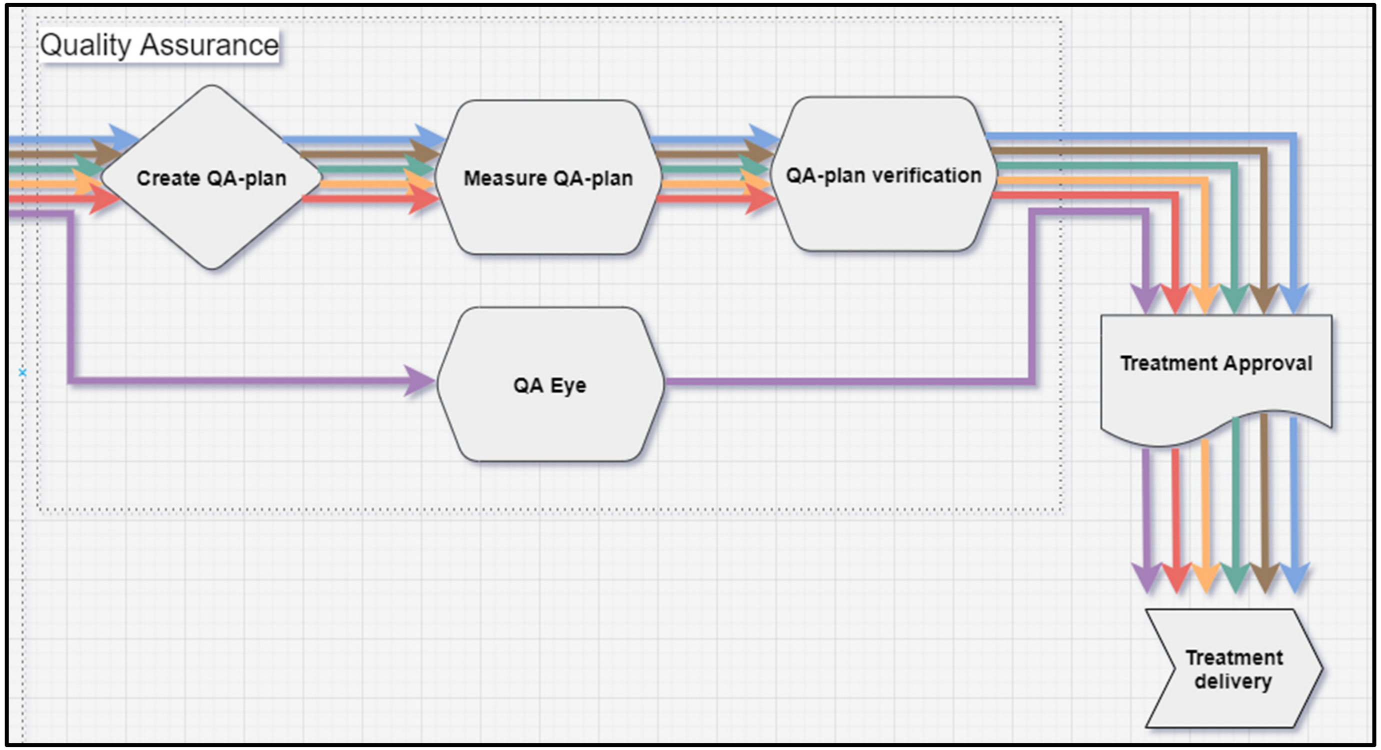

Treatment delivery, treatment plan adaptation and follow up

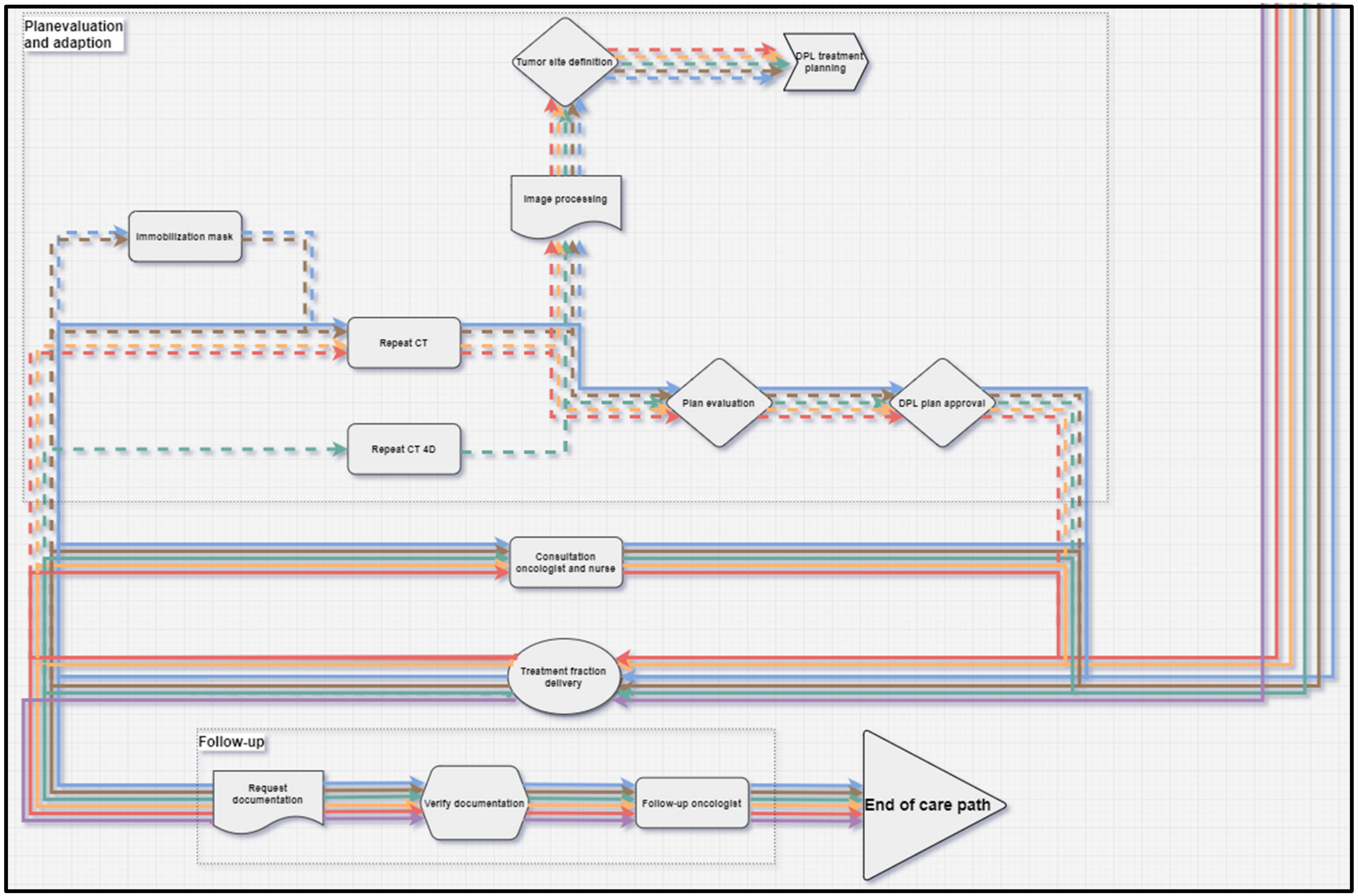

Supplementary Materials S2: Head & neck

Preparation phase, in minutes

Care path activities	Physician	Nurse	Treatment imaging RTT	Treatment planning RTT	Physicist	Medical physics engineer	Estimation method
Treatment planning for comparison				45			Time recording
Approve treatment plan	25						Interview
Plan comparison meeting	10*			5	5		Interview
Treatment plan check				10	30		Interview
Phone consultation		15					R&V system
First consultation + Endoscopy	45	60					Interview
Overall administration	40						Interview
Set-up meeting	5		**				Interview
Immobilization mask			**				R&V system
Contrast infuse		10					R&V system
CT planning			90*				R&V system
MRI planning			120*				R&V system
PET planning			200*				R&V system
Image processing				515			Time recording
CTV definition	240						Interview

**Second check definition**	60*						Interview
**Treatment planning**				10			Time recording
**Treatment plan check**				10			Interview
**Approve treatment plan**	30						Interview
**Treatment plan meeting**	20*			10	10		Interview
**Phone consultation**		15					Time recording
**Treatment plan processing**				10			Interview
**Plan processing check**				5			Interview
**Treatment plan check**					30		Interview
**Create QA plan**						30	Interview
**Measure QA plan**						80*	Time recording
**Check QA plan**					2.5		Interview
**Treatment approval**					2.5		Interview
**TOTAL (excl. PET)**	**475**	**100**	**210**	**620**	**80**	**110**	

* Summed time estimate based on two participating employees.

** Time estimate is included in the "CT planning" task.

N.B: The "PET planning" care path activity, depicted in darker green, is optional.

Treatment and follow-up phase (in minutes)

Care path activities	Physician	Nurse	Treatment imaging RTT	Treatment planning RTT	Treatment delivery RTT	Dietician	Estimation method
**Treatment delivery***					30 min · 35 · 3 RTT's = 3150		R&V system
**Consultation during treatment****	15 · 6 = 90	20 · 6 = 120				45 · 6 =270	Interview and R&V system
**CT repetition*****			15 · 4 · 2 = 120				R&V system
**Plan evaluation*****				90 · 4 = 360			Interview
**Plan approval*****	15 · 4 = 60						Interview
**Follow-up at XXXX**	20 · 4 = 80 (After 6 weeks, 3 months, 6 months, 12 months)	20 · 2 = 40 (After 1st week and after 3 weeks)	120 (MRI after 3 months)				Interview
**TOTAL**	**230**	**160**	**240**	**360**	**3150**	**270**	

* Each treatment fraction is executed by 3 RTTs each day.

** Summed estimate, based on weekly consultations from the 2nd week of treatment delivery (6 in total).

*** Summed estimate, based on 4 evaluation CT scans during the treatment phase.

Total human resource consumption (in minutes)

	Physician	Nurse	Treatment imaging RTT	Treatment planning RTT	Physicist	Medical physics engineer	Treatment delivery RTT	Dietician
**Preparation phase**	475	100	210	620	80	110	-	-
**Treatment and follow-up phase**	230	160	240	360	-	-	3150	270
**TOTAL**	**705**	**260**	**450**	**980**	**80**	**110**	**3150**	270

Available fractionation schemes

Fractionation schemes			
Oropharynx	Nasopharynx	Larynx	Hypopharynx
**35** x 2Gy = 70Gy	**35** x 2Gy = 70Gy	**35** x 2Gy = 70Gy	**35** x 2Gy = 70Gy

Supplementary Materials S3: Brain

Preparation phase (in minutes)

**Care path activities**	**Physician**	**Nurse**	**Treatment imaging RTT**	**Treatment planning RTT**	**Physics group**	**Medical physics engineer**	**Estimation method**
Treatment planning for comparison				45			Time recording
Approve treatment plan	25						Interview
Plan comparison meeting	10*			5	5		Interview
Treatment plan check				10	30		Interview
**Overall administration**	40						Interview
**Phone consultation**		15					R&V system
**First consultation**	45	45					Interview
**Set-up meeting**	5		**				Interview
**Immobilization mask**			**				R&V system
**Contrast infuse**		10					R&V system
**CT planning**			80*				R&V system
**MRI planning**			120*				R&V system
**Image processing**				395			Time recording
**CTV definition**	150						Interview
**Second check definition**	60*						Interview
**Dry-run gantry**				15			Interview

**Treatment planning**				30			Time recording
**Treatment plan check**				10			Interview
**Approve treatment plan**	30						Interview
**Treatment plan meeting**	20*			10	10		Interview
**Phone consultation**		15					Time recording
**Treatment plan processing**				10			Interview
**Plan processing check**				5			Interview
**Treatment plan check**					30		Interview
**Create QA plan**						30	Interview
**Measure QA plan**						60*	Time recording
**Check QA plan**					2.5		Interview
**Treatment approval**					2.5		Interview
**TOTAL**	**385**	**85**	**200**	**535**	**80**	**90**	

* Summed time estimate based on two participating employees.

** Time estimate is included in the "CT planning" task.

Treatment and follow-up phase (in minutes)

Care path activities	Physician	Treatment delivery RTT	Estimation method
**Treatment delivery***		(28 fraction scheme)<br>20 min · 28 · 3 RTT's =<br>1680	R&V system

		(33 fraction scheme)<br>20 min · 33 · 3 RTT's =<br>1980	
**Consult during treatment****	(28 fraction scheme)<br>5 · 15 = 75<br>(33 fraction scheme)<br>6 · 15 = 90		Interview
**Follow-up**<br>**(phone consultation)**	3 · 15 = 45<br>(After 1 month, 6 months and 1 year)		Interview
**TOTAL (28 fraction scheme)**	**120**	**1680**	
**TOTAL (33 fraction scheme)**	**135**	**1980**	

* Each treatment fraction is executed by 3 RTTs each day

** Summed estimate, based on weekly consultations

Total human resource consumption (in minutes)

	Physician	Nurse	Treatment imaging RTT	Treatment planning RTT	Physicist	Medical physics engineer	Treatment delivery RTT
**Preparation phase**	385	85	200	535	80	90	
**Treatment and follow-up phase (28 fraction scheme)**	120	-	-	-	-	-	1680

**Treatment and follow-up phase (33 fraction scheme)**	135	-	-	-	-	-	1980
**TOTAL (28 fraction scheme)**	**505**	**85**	**200**	**535**	**80**	**90**	**1680**
**TOTAL (33 fraction scheme)**	**520**	**85**	**200**	**535**	**80**	**90**	**1980**

Available fractionation schemes

**Fractionation schemes**				
Meningeoma	Low grade glioma	Anaplastic glioma	Hyopfyse	Craniofayngioma
Benigne: **30** x 1.8Gy = 54Gy<br>Atypical: **30** x 2.0Gy = 60Gy	**28** x 1.8Gy = 50.4Gy	**33** x 1.8Gy = 59.4Gy	**28** x 1.8Gy = 50.4Gy	**30** x 1.8Gy = 54Gy

Supplementary Materials S4: Breast

Preparation phase (in minutes)

Care path activities	Physician	Nurse	Treatment imaging RTT	Treatment planning RTT	Physics group	Medical physics engineer	Estimation method
Treatment planning for comparison				35			Time recording
Approve treatment plan	25						Interview
Plan comparison meeting	10*			5	5		Interview
Treatment plan check				10	30		Interview
**Phone consultation**		15					Time recording
**First consultation**	45	45					Interview

**Overall administration**	40						Interview
**Set-up meeting**	5		**				Interview
**CT planning**			80*				R&V system
**Dry-run gantry**			30*				R&V system
**Image processing**				50			Time recording
**CTV definition**	150						Interview
**Second check definition**	40*						Interview
**Treatment planning**				10			Time recording
**Treatment plan check**				10			Interview
**Approve treatment plan**	30						Interview
**Treatment plan meeting**	20*			10	10		Interview
**Phone consultation**		15					Time recording
**Treatment plan processing**				10			Interview
**Plan processing check**				5			Interview
**Treatment plan check**					30		Interview
**Create QA plan**						30	Interview
**Measure QA plan**						60*	Time recording
**Check QA plan**					2.5		Interview
**Treatment approval**					2.5		Interview
**TOTAL**	**395**	**75**	**110**	**145**	**80**	**90**	

* Summed time estimate based on two participating employees.

** Time estimate is included in the “CT planning” task.

Treatment and follow-up phase (in minutes)

Care path activities	Physician	Nurse	Treatment delivery RTT	Estimation method
**Treatment delivery***			(15 fraction scheme)<br>30 min · 15 · 3 RTT's = 1350<br>(22 fraction scheme)<br>30 min · 22 · 3 RTT's = 1980	R&V system
**Consult during treatment****	(15 fraction scheme)<br>2 · 15 = 30<br>(22 fraction scheme)<br>3 · 15 = 45	(15 fraction scheme)<br>3 · 15 = 45<br>(22 fraction scheme)<br>4 · 15 = 60		Interview
**Follow-up at XXXX**	4 · 20 = 80<br>(After 2 weeks<br>3 months, 6 months and 1 year)			Interview
**TOTAL<br>(15 fraction scheme)**	**110**	**45**	**1350**	
**TOTAL<br>(22 fraction scheme)**	**125**	**60**	**1980**	

* Each treatment fraction is executed by 3 RTTs each day

** Summed estimate, based on weekly consultations from the 2nd week of treatment delivery.

Total human resource consumption (in minutes)

	Physician	Nurse	Treatment imaging RTT	Treatment planning RTT	Physicist	Medical physics engineer	Treatment delivery RTT
**Preparation phase**	395	75	110	145	80	90	
**Treatment and follow-up phase (15 fraction scheme)**	110	45	-	-	-	-	1350
**Treatment and follow-up phase (22 fraction scheme)**	125	60	-	-	-	-	1980
**TOTAL (15 fraction scheme)**	**505**	**120**	**110**	**145**	**80**	**90**	**1350**
**TOTAL (22 fraction scheme)**	**520**	**135**	**110**	**145**	**80**	**90**	**1980**

Available fractionation schemes

Fractionation schemes		
**15** x 2.67Gy = 40.05Gy	**20** x 2.67Gy = 53.40Gy	**22** x 2.67Gy = 58.74Gy

Supplementary Materials S5: Thorax (lymphoma and lung)

Preparation phase (in minutes)

Care path activities	Physician	Nurse	Treatment imaging RTT	Treatment planning RTT	Physics group	Medical physics engineer	Estimation method

Treatment planning for comparison				105			Time recording
Approve treatment plan	25						Interview
Plan comparison meeting	10*			5	5		Interview
Treatment plan check				10	30		Interview
Overall administration	40						Interview
Phone consultation		15					Time recording
First consultation	45	45					Interview
Set-up meeting	5		**				Interview
Contrast infuse		10					R&V system
CT 4D planning			80*				R&V system
PET planning			200*				R&V system
Dry-run gantry			30*				R&V system
Image processing				120			Time recording
CTV definition	180						Interview
Second check definition	60*						Interview
Treatment planning				15			Time recording
Treatment plan check				10			Interview
Approve treatment plan	30						Interview
Treatment plan meeting	20*			10	10		Interview
Phone consultation		15					Time recording
Treatment plan processing				10			Interview

**Plan processing check**				5			Interview
**Treatment plan check**					30		Interview
**Create QA plan**						30	Interview
**Measure QA plan**						70*	Time recording
**Check QA plan**					2.5		Interview
**Treatment approval**					2.5		Interview
**TOTAL**	**415**	**85**	**110**	**290**	**80**	**100**	

* Summed time estimate based on two participating employees.

** Time estimate is included in the “CT planning” task.

N.B: The “PET planning” care path activity, depicted in darker green, is optional.

Treatment and follow-up phase (in minutes)

Care path activities	Physician	Nurse	Treatment delivery RTT	Estimation method
**Treatment delivery***			(15 fraction scheme)<br>25 min · 15 · 3 RTT’s = 1125<br>(30 fraction scheme)<br>25 min · 30 · 3 RTT’s = 2250	R&V system
**Consult during treatment****	(15 fraction scheme)<br>3 · 15 = 45<br>(30 fraction scheme)<br>6 · 15 = 90	(30 fraction scheme)<br>6 · 15 = 90		Interview

**Follow-up at XXXX**	20<br>(End of treatment)			Interview
**TOTAL**<br>**(15 fraction scheme)**	**65**	**-**	**1125**	
**TOTAL**<br>**(30 fraction scheme)**	**110**	**90*** **	**2250**	

* Each treatment fraction is executed by 3 RTTs each day

** Summed estimate, based on weekly consultations

*** Nurse consultations during the treatment phase is not conducted for lymphoma patients (15 and 18 fractionation schemes).

Total human resource consumption (in minutes)

	Physician	Nurse	Treatment imaging RTT	Treatment planning RTT	Physicist	Medical physics engineer	Treatment delivery RTT
**Preparation phase**	415	85	110	290	80	100	
**Treatment and follow-up phase (15 fraction scheme)**	65	-			-	-	1125
**Treatment and follow-up phase (30 fraction scheme)**	110	90			-	-	2250
**TOTAL (15 fraction scheme)**	**480**	**85**	**110**	**290**	**80**	**100**	**1125**
**TOTAL (30 fraction scheme)**	**525**	**175**	**110**	**290**	**80**	**100**	**2250**

Available fractionation schemes

Fractionation schemes			
Hodgkin Lymphoma		Non-Hodgkin Lymphoma	
Early stage <60 year	Advanced stage <60 year	Stage 1/2	Stage 3/4
**15** x 2Gy = 30Gy	**18** x 2Gy = 36Gy	**18** x 2Gy = 36Gy	**15** x 2Gy = 30Gy<br>**18** x 2Gy = 36Gy
Non-small cell lung cancer	Small cell lung cancer	Thymoma	Sarcoma
**30** x 2Gy = 60Gy<br>**25** x 2Gy = 50Gy<br>**30** x 2Gy = 60Gy	**30** x 1.5Gy = 45Gy<br>**25** x 2 = 50Gy	**25** x 2 = 50Gy<br>**30** x 2 = 60Gy	**25** x 2 = 50Gy

Supplementary Materials S6: Chordoma

Preparation phase (in minutes)

Care path activities	Physician	Nurse	Treatment imaging RTT	Treatment planning RTT	Physics group	Medical physics engineer	Estimation method
**Phone consultation**		15					Time recording
**First consultation**	45	45					Interview
**Overall administration**	40						Interview
**Set-up meeting**	5		**				Interview
**Contrast infuse**		10					R&V system
**CT planning**			80*				R&V system
**MRI planning**			120*				R&V system
**Image processing**				155 (515)***			Time recording
**CTV definition**	180 (360)***						Interview
**Second check definition**	60*						Interview
**Treatment planning**				10 (480)***			Time recording
**Treatment plan check**				10			Interview
**Approve treatment plan**	30						Interview
**Treatment plan meeting**	20*			10	10		Interview

**Phone consultation**		15					Time recording
**Treatment plan processing**				10			Interview
**Plan processing check**				5			Interview
**Treatment plan check**					30<br>(60)***		Interview
**Create QA plan**						30	Interview
**Measure QA plan**						80*	Time recording
**Check QA plan**					2.5		Interview
**Treatment approval**					2.5		Interview
**TOTAL (spinal column)**	**380**	**85**	**200**	**200**	**45**	**110**	
**TOTAL (skull base)**	**560**	**85**	**200**	**1030**	**75**	**110**	

* Summed time estimate based on two participating employees.

** Time estimate is included in the "CT planning" task.

***Estimated minutes in parenthesis is particularly for Skull base chordoma's.

Treatment and follow-up phase (in minutes)

Care path activities	Physician	Treatment delivery RTT	Estimation method
**Treatment delivery***		(35 fraction scheme)<br>25 min · 35 · 3 RTT's = 2625<br><br>(37 fraction scheme)<br>25 min · 37 · 3 RTT's = 2775	R&V system

**Consult during treatment****	(35 fraction scheme)<br>15 · 7 = 105<br><br>(37 fraction scheme)<br>15 · 7 = 105		Interview
**Follow-up at XXXX**	20<br>(After 1 month)		Interview
**TOTAL (35 fraction scheme)**	**125**	**2625**	
**TOTAL (37 fraction scheme)**	**125**	**2775**	

* Each treatment fraction is executed by 3 RTTs each day

** Summed estimate, based on weekly consultations

Total human resource consumption (in minutes)

	Physician	Nurse	Treatment imaging RTT	Treatment planning RTT	Physicist	Medical physics engineer	Treatment delivery RTT
**Preparation phase**	380	85	200	200	45	110	
**Preparation phase (skull base)**	560	85	200	1030	75	110	
**Treatment and follow-up phase (35 fraction scheme)**	125	-	-	-	-	-	2625
**Treatment and follow-up phase (37 fraction scheme)**	125	-	-	-	-	-	2775

**TOTAL for spinal column chordoma's (35 fraction scheme)**	**505**	**85**	**200**	**200**	**45**	**110**	**2625**
**TOTAL for spinal column chordoma's (37 fraction scheme)**	**505**	**85**	**200**	**200**	**45**	**110**	**2775**
**TOTAL for skull base chordoma's (35 fraction scheme)**	**685**	**85**	**200**	**1030**	**75**	**110**	**2625**
**TOTAL for skull base chordoma's (37 fraction scheme)**	**685**	**85**	**200**	**1030**	**75**	**110**	**2775**

Available fractionation schemes

**Fractionation schemes**	
**Chordoma (spinal column)**	**Skull base chordoma**
Post-surgery and primary: **35** x 2Gy = 70Gy	Post-surgery and primary: **35** x 2Gy = 70Gy
Post-surgery and primary: **37** x 2Gy = 74Gy	Post-surgery and primary: **37** x 2Gy = 74Gy

Supplementary Materials S7: Eye melanoma

Preparation phase (in minutes)

Care path activities	Physician	Nurse	Treatment imaging RTT	Treatment planning RTT	Physics group	Medical physics engineer	Estimation method
Multicenter eye patient meeting	20*						Interview
Phone consultation		15					Time recording
First consultation	45	15					Interview
Overall administration	30						Interview
Set-up meeting	5		10*				Interview
Immobilization mask			60*				Interview
Eye simulation			90*				Interview
Image processing eye			***				Interview
CTV definition	240*						Interview
Treatment planning				150*			Interview
Approve treatment plan	**						Interview
Treatment plan meeting	20*						Interview
Create aperture						30*	Interview
Dry run				70*			Interview
Treatment plan check					30		Interview
QA eye						90*	Interview

**Treatment approval**					5		Interview
**TOTAL**	**360**	**30**	**160**	**220**	**35**	**110**	

* Summed time estimate based on two participating employees.

** Time estimate is included in the “CTV definition” task.

*** Time estimate is included in the “Eye simulation” task.

Treatment and follow-up phase (in minutes)

	Physician	Treatment delivery RTT	Estimation method
**Treatment delivery***	40 min · 4 = 160	40 min · 4 · 2 RTT’s = 320	Interview
**Consult during treatment****	25		Interview
**Follow-up (phone consultation)**	15 · 3 = 45 (End of treatment, after 1 month and after 1 year)		Interview
**TOTAL**	**240**	**320**	

*Each treatment fraction is executed by 2 RTTs and a physician each day

** Once during the treatment phase

Total human resource consumption (in minutes)

	Physician	Nurse	Treatment imaging RTT	Treatment planning RTT	Physicist	Medical physics engineer	Treatment delivery RTT
**Preparation phase**	360	30	160	220	35	110	-
**Treatment and follow-up phase**	240	-	-	-	-	-	320

**TOTAL**	**600**	**30**	**160**	**220**	**35**	**110**	**320**

Available fractionation scheme

Fractionation scheme
**4** x 15Gy = 60Gy

Supplementary Materials S8: Treatment plan adaptation (excluding eye melanoma)

Adaptation phase (in minutes)

Care path activities	Physician	Treatment imaging RTT	Treatment planning RTT	Physics group	Medical physics engineer	Estimation method
**Immobilization mask**		10*				R&V system
**CT planning**		80*				R&V system
**Image processing**			***			Interview
**CTV definition**	30 to 90**					Interview
**Treatment planning**			60			Interview
**Treatment plan check**			***			Interview
**Approve treatment plan**	30					Interview
**Treatment plan meeting**	20*		***	10		Interview
**Treatment plan processing**			***			Interview
**Plan processing check**			***			Interview
**Treatment plan check**				20		Interview
**Create QA plan**					30	Interview
**Measure QA plan**					60*	Time recording
**Check QA plan**				2.5		Interview
**Treatment approval**				2.5		Interview
**TOTAL (breast)**	**80**	**90**	**60**	**35**	**90**	

**Average per patient (p = 10%)**	**8**	**9**	**6**	**3.5**	**9**	
**TOTAL (head & neck)**	**140**	**90**	**60**	**35**	**90**	
**Average per patient (p = 80%)**	**112**	**72**	**48**	**28**	**72**	

* Summed time estimate based on two participating employees.

** CTV definition varies between 30 minutes for breast plan adaptations and 90 minutes for head & neck plan adaptations.

*** Time estimates are included in the “Treatment planning” task.
